# Seamless meningioma resection in a patient with coexisting myoclonic epilepsy and sporadic Pick’s disease

**DOI:** 10.1093/omcr/omaf116

**Published:** 2025-07-27

**Authors:** Jose Maaz, Carlos Diaz, Marcos Orellana, Ricardo A Caravantes

**Affiliations:** Department of Medical Research, Universidad Francisco Marroquin, 6ta Calle Final Zona 10, Guatemala City 01010, Guatemala; Department of Medical Research, Universidad Francisco Marroquin, 6ta Calle Final Zona 10, Guatemala City 01010, Guatemala; Department of Medical Research, Universidad Francisco Marroquin, 6ta Calle Final Zona 10, Guatemala City 01010, Guatemala; Department of Medical Research, Universidad Francisco Marroquin, 6ta Calle Final Zona 10, Guatemala City 01010, Guatemala

**Keywords:** syncope episodes, neurological symptoms, hypertension, unsteady gait, ataxic gait, cerebellar involvement, myoclonic epilepsy, sporadic Pick’s disease

## Abstract

We report the case of a 62-year-old male presenting with progressive gait disturbances, recurrent syncope, and episodes of myoclonic epilepsy over three months, with underlying sporadic Pick’s disease diagnosed clinically. Imaging revealed a well-defined, extra-axial tumor in the right parietal region, consistent with a meningioma. Surgical intervention using neuronavigation technology facilitated a complete tumor resection. The patient’s postoperative course was stable, with a resolution of syncope and improvement in gait. This case underscores the importance of interdisciplinary management in patients with overlapping neurological conditions and highlights the challenges posed by multifaceted clinical presentations.

## Introduction

Intracranial tumors such as meningiomas often present with diverse symptoms depending on their size and location. In rare instances, these symptoms may coexist with other unrelated but compounding neurological conditions, creating diagnostic and therapeutic challenges. Meningiomas are rare in children and young adults; they represent 1%–3% of all intracranial tumors in individuals up to age 20 years and 13.5% of intracranial tumors in the 20–34 age group. Meningioma in young adult male is a rare finding [1]. Here, we describe a 62-year-old male with a right parietal meningioma, coexisting myoclonic epilepsy, and clinically diagnosed sporadic Pick’s disease. Pick disease is uncommon and accounts for less than 2% of adult-onset dementias [2]. This unique case underscores the importance of timely intervention in complex presentations involving multiple neurological conditions.

## Case presentation

A 62-year-old male presented with a puzzling three-month history of worsening neurological symptoms. Initial complaints of mild ataxia progressed to frequent episodes of syncope and unsteadiness. Family members reported recent changes in the patient’s personality, including emotional blunting and repetitive behaviors, raising concerns of an underlying neurodegenerative disorder.

The patient had a history of untreated hypertension but no prior surgeries, trauma, or significant family history of neurological disease. Physical examination revealed unsteady, ataxic gait and mild dysarthria. Neurological examination detected reduced coordination and episodic myoclonic jerks involving the upper limbs. His Mini-Mental State Examination (MMSE) score was 24/30, with impairments in executive function and memory, consistent with early frontotemporal dementia.

A cardiovascular workup, including ECG and Holter monitoring, was unremarkable, ruling out cardiac causes for syncope. A CT scan revealed a large, extra-axial mass in the right parietal region, consistent with a meningioma. Advanced MRI confirmed compression of adjacent cortical structures, correlating with his symptoms.

The diagnosis of sporadic Pick’s disease was made clinically based on personality changes, executive dysfunction, and behavioral symptoms, supported by imaging findings of frontotemporal atrophy on MRI.

**Figure 1:**
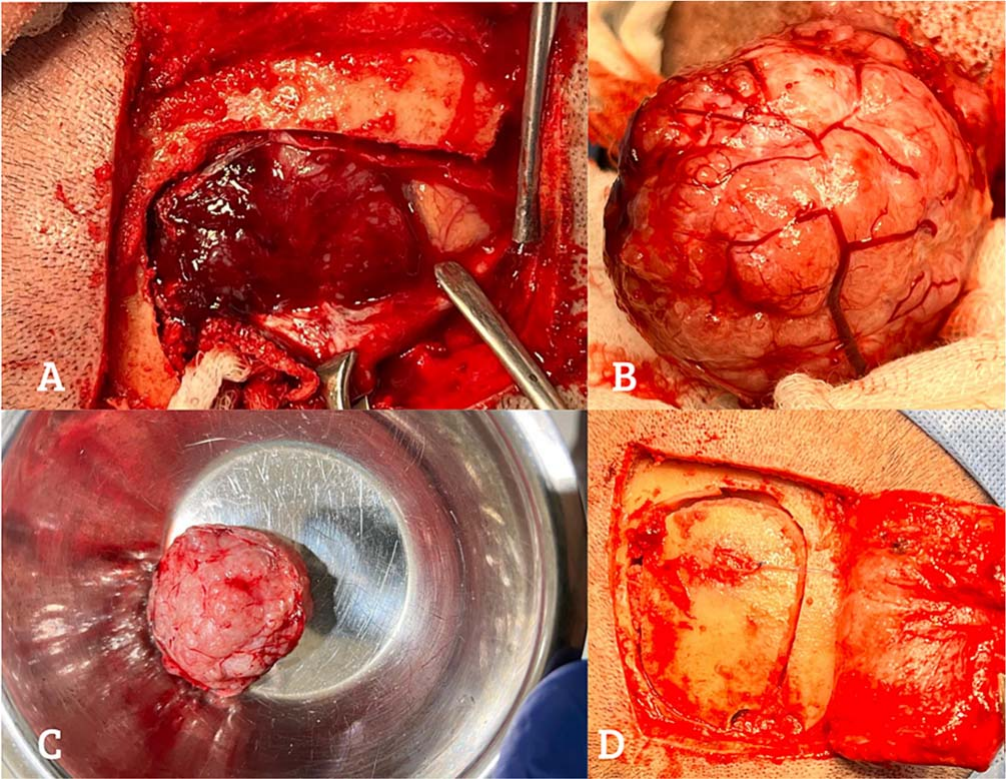
Intraoperative stages of a craniotomy for the resection of an extra-axial meningioma. (A) Shows the exposure of a well-defined mass in the right parietal region. (B) Displays the extraction of the meningioma out of the intracranial cavity, preserving the integrity of surrounding structures. (C) Presents the fully resected meningioma, displaying its characteristic macroscopic features such as a firm texture and gray-white color. (D) Shows a post-craniotomy view.

**Figure 2:**
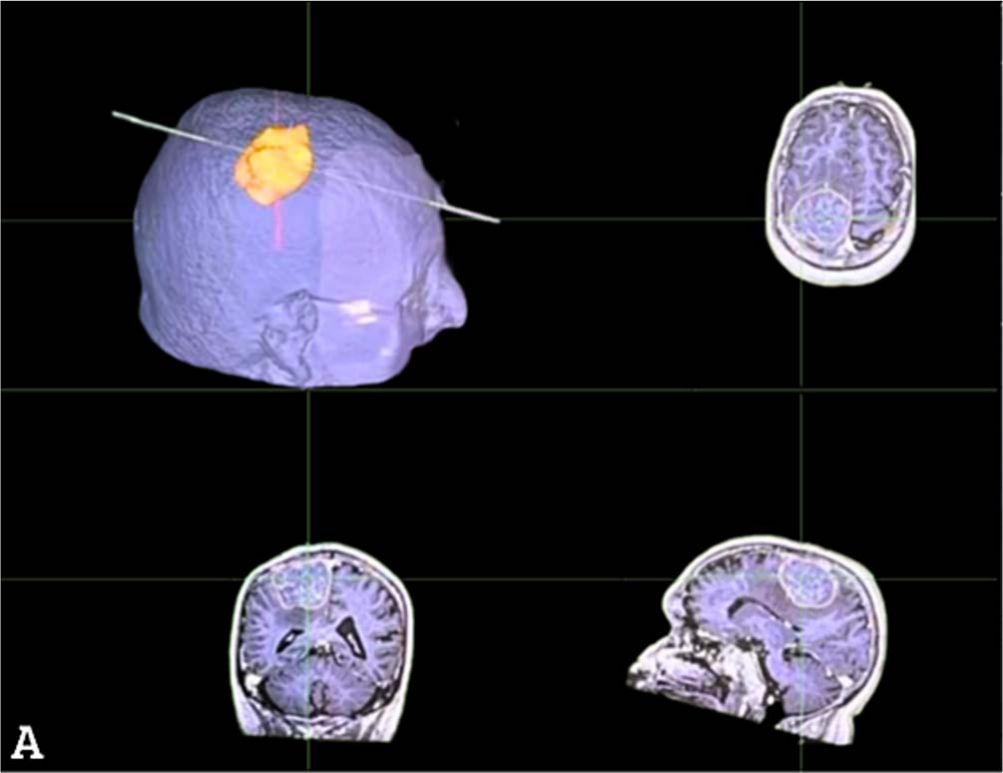
Perioperative neuronavigation. Display MRI sequences with axial, coronal and sagittal views and a 3D reconstruction of the tumor in relation to the skull aiding in surgical planning and demonstrating the tumor’s size.

The tumor’s size and location necessitated immediate surgical resection. Using neuronavigation, a 6x6 cm firm, grayish-white meningioma was excised without complications. Interestingly, intraoperative findings revealed a highly vascularized tumor, adding complexity to the procedure [Figs 1 and 2].

Lesions in the parietal region can cause a range of symptoms due to its role in sensory and motor integration. In this case, the right parietal meningioma likely impacted both the primary and secondary motor areas, leading to gait dysfunction. Its proximity to the primary motor and somatosensory cortices may have caused ataxia, gait instability, and syncope. The tumor’s location may have also interfered with the connection between the motor cortex and cerebellum, contributing to these symptoms. Histologically, it was a Grade I meningioma with a well-defined, firm appearance, and vascular features typical of angiomatous meningiomas, which can complicate surgical resection due to their rich blood supply.

Postoperatively, the patient’s syncope episodes resolved, his gait improved significantly, and his personality changes stabilized. The myoclonic epilepsy episodes persisted but were controlled with valproic acid, with adjustments made in collaboration with a neurologist specializing in epilepsy.

The patient was regularly followed up after meningioma resection at 1, 3, and 6 months, showing significant improvement in gait and resolution of syncope episodes. Myoclonic jerks were well-controlled with adjusted antiepileptic treatment. Cognitive function, assessed with the Mini-Mental State Examination (MMSE), showed mild cognitive impairment initially (24/30), with improvement to 26/30 at 3 and 6 months, suggesting partial stabilization of cognitive decline. The Montreal Cognitive Assessment (MoCA) remained stable. These findings indicate that the meningioma resection helped stabilize cognitive symptoms, although the cognitive decline from Pick’s disease showed no significant changes.

## Discussion

The co-occurrence of a meningioma, myoclonic epilepsy, and clinically diagnosed sporadic Pick’s disease presents a unique and challenging clinical scenario. While these conditions have distinct etiologies, their overlapping symptoms can complicate diagnosis and management, necessitating a comprehensive approach [3].

The majority of meningiomas grow slowly and are histologically benign, making them potentially curable through surgery alone. Symptoms vary depending on the tumor’s location, often presenting as signs of mass effect or neurological impairments. Seizures, which occur in about 30% of cases, are a common initial symptom that significantly impact quality of life, reduce independence, hinder cognitive abilities, and elevate the risk of psychiatric conditions such as depression. While surgical removal can achieve seizure control in 60%–90% of cases, approximately 12%–19% of patients experience persistent seizures post-surgery [4].

Meningiomas, originating from arachnoid cap cells, are commonly slow-growing extra-axial tumors. However, their clinical impact is determined by size, location, and the degree of mass effect on adjacent structures. In this case, the tumor’s parietal location and proximity to motor and sensory cortices explained the patient’s progressive gait disturbances and syncope [5]. Notably, the tumor’s unusual vascularization likely contributed to the rapid onset of severe symptoms. This vascular characteristic, while uncommon in meningiomas, is described in specific subtypes, such as angiomatous meningiomas, which warrant further histopathological correlation [6].

The patient’s myoclonic epilepsy likely resulted from cortical irritation caused by the tumor’s compression of adjacent brain structures [7]. Seizures in the context of meningiomas are a well-documented phenomenon, particularly in tumors involving motor or sensory areas. Effective management requires surgical removal of the tumor, followed by tailored antiepileptic therapy. In this case, valproic acid effectively controlled the residual epileptic activity post-resection [8, 9].

The diagnosis of sporadic Pick’s disease added an additional layer of complexity to this case. While unrelated to the meningioma, frontotemporal atrophy associated with Pick’s disease can independently manifest as personality changes, executive dysfunction, and cognitive decline. These symptoms may mimic or amplify the neurological deficits caused by the meningioma, further emphasizing the importance of distinguishing between the two conditions during clinical evaluation [10]. The diagnosis of Pick’s disease in this patient was based on clinical features and imaging findings, as genetic testing and histopathological confirmation are not routinely performed in sporadic cases.

Surgical management of this case was guided by neuronavigation, a cornerstone in modern neurosurgery. The integration of preoperative imaging with intraoperative guidance ensures precise resection while minimizing damage to critical brain regions. Neuronavigation was essential in delineating the tumor’s borders, particularly given its vascular nature and proximity to the motor cortex. Complete resection of the tumor significantly alleviated the patient’s symptoms, highlighting the efficacy of surgical intervention in resolving neurological deficits caused by meningiomas.

The patient was diagnosed with myoclonic epilepsy based on a clinical history of episodic myoclonic jerks and other neurological symptoms, despite the absence of an EEG or genetic testing. The myoclonic jerks responded to valproic acid, supporting the diagnosis. Genetic testing was not pursued due to the lack of clear genetic indicators. The patient’s seizures were effectively managed through collaboration with an epilepsy specialist. This case highlights the importance of a multidisciplinary approach in managing overlapping neurological conditions, addressing both the primary meningioma and coexisting epilepsy and neurodegenerative disease for optimal outcomes.

The coexistence of a parietal meningioma, seizures, and sporadic Pick’s disease in this patient created diagnostic challenges. The tumor likely caused focal seizures, but myoclonic jerks in both upper limbs suggest possible secondary generalization. Symptoms such as gait instability and cognitive fog pointed toward bvFTD, which was confirmed by postoperative FDG-PET showing hypometabolism in the frontal and anterior temporal lobes. This case highlights the complexity of overlapping neurological disorders and the importance of a comprehensive neurodiagnostic approach to clarify the clinical picture and guide treatment.

## Conclusion

This case underscores the importance of considering overlapping neurological diagnoses in patients with complex clinical presentations. Early recognition and surgical intervention for the meningioma, coupled with symptomatic management of coexisting conditions, led to a favorable outcome. Long-term follow-up is essential to monitor the progression of Pick’s disease and manage residual epilepsy. This case demonstrates the value of a multidisciplinary approach in achieving optimal outcomes for patients with multifactorial neurological conditions.
